# The effectiveness of virtual reality-based exercises in patients with symptomatic knee osteoarthritis: randomized controlled study

**DOI:** 10.1590/1806-9282.20260294

**Published:** 2026-07-24

**Authors:** Basak Cigdem-Karacay, Seyda-Sultan Eraslan, İrem Canli, Atahan Turhan

**Affiliations:** 1 Kirsehir Ahi Evran University, Faculty of Medicine, Department of Physical Medicine and Rehabilitation – Kırşehir, Turkey.; 2 Kirsehir Ahi Evran University, School of Physiotherapy and Rehabilitation – Kırşehir, Turkey.; 3 Kirsehir Ahi Evran University, Rectorship, Pilot University Coordinatorship of Health – Kırşehir, Turkey.

**Keywords:** Virtual reality, Knee osteoarthritis, Exercise therapy, Quality of life, Chronic pain

## Abstract

**OBJECTIVE:**

The aim of this study was to investigate the effectiveness of adding non-immersive virtual reality-based exercise to a conventional rehabilitation program for pain, functionality, balance, and quality of life in patients with knee osteoarthritis.

**METHODS:**

This randomized controlled clinical trial involved 60 patients. Measurements were taken before and after treatment (at week 3). Visual analog scale, Western Ontario and McMaster Universities Osteoarthritis Index, and the Short Form-36 were measured. Postural Stability– Dynamic Overall Stability Index, Functional Reach Composite Score, and Clinical Test of Sensory Interaction on Balance–Composite Score were recorded using the Biodex Balance System.

**RESULTS:**

In both groups, statistically significant improvements were observed in visual analog scale, Western Ontario and McMaster Universities Osteoarthritis Index, the Short Form-36 Physical Functioning, Postural Stability–Dynamic Overall Stability Index, Functional Reach Composite Score, and Clinical Test of Sensory Interaction on Balance–Composite Score scores in the post-intervention period (p<0.001). In both groups, statistically significant improvements were observed in all Short Form-36 subscales [Physical Functioning, Role Physical, Bodily Pain, General Health, Vitality, Social Functioning, Role Emotional, and Mental Health were observed during the post-intervention period (p<0.001)]. In the two-way mixed-methods repeated-measures analysis of variance analysis, the time effect was found to be significant in all Short Form-36 subdimensions (p<0.001). The group-time interaction was not significant in the “Role Physical” and “Mental Health” subdimensions (p=0.151 and p=0.604, respectively).

**CONCLUSION:**

The addition of virtual reality-based exercise to results in greater reductions in pain levels and improvements in disease-specific functional status and overall physical function, postural stability, dynamic balance, and sensory integration. Further improvements in quality of life across all subscales except for physical role limitations and mental health.

## INTRODUCTION

Knee osteoarthritis is a significant health problem that affects quality of life by causing pain and functional limitations^1^. The cornerstone of conventional treatment for knee osteoarthritis is patient-specific quadriceps strengthening exercises^2,3^.

Virtual reality-based exercises aim to perform repetitive movements safely, controllably, and immersively through games that resemble daily living activities, with the support of technology. Research on the use of virtual reality-based exercises in rehabilitation has accelerated in recent years^4^. The effectiveness of virtual reality-based exercise methods in the rehabilitation of stroke, multiple sclerosis, Parkinson’s disease, and cerebral palsy has been investigated^5,6^. In addition to neurological rehabilitation, the effectiveness of virtual reality-based exercise has been investigated for cancer-related dysfunctions, cardiac rehabilitation, and vestibular rehabilitation. It has been reported that virtual reality-based exercises improve mobility by reducing kinesiophobia in patients with chronic pain^4^. Kinesiophobia is associated with pain, functional limitations, and mobility restrictions in patients with knee osteoarthritis, negatively impacting exercise adherence and rehabilitation^7^. Virtual reality-based video games increase participation in exercise and create an immersive environment characterized by repetitive movements^4,6^.

Virtual reality-based exercises can be implemented using either an immersive method, in which the individual is fully immersed in the virtual environment, or a non-immersive method, in which the individual interacts only through a screen as an external observer^8,9,10,11^. The effectiveness of both immersive and non-immersive virtual reality-based rehabilitation in chronic musculoskeletal diseases has been reported^12^.

Recent meta-analyses examining the effectiveness of virtual reality-based exercises in knee osteoarthritis have reported that such interventions may be effective in reducing pain and improving muscle strength; however, the number of studies is limited, and further research is needed in patients with knee osteoarthritis, particularly to support clinical application^13,14^.

Most studies in the literature examining the effectiveness of virtual reality-based exercise therapy in knee osteoarthritis rehabilitation have focused on pain and function^13^. A few studies have assessed balance and proprioception without the use of devices^15^. To the authors’ knowledge, there are no research examining the effectiveness of virtual reality-based exercise therapy on quality of life in patients with knee osteoarthritis has been found in the literature. This study aimed to investigate the short-term effectiveness of non-immersive virtual reality-based exercises, added to a conventional rehabilitation program, on pain, function, balance, and quality of life in patients with symptomatic knee osteoarthritis.

## METHODS

### Study population and design

This randomized controlled single-blinded clinical trial involved 60 patients with symptomatic knee osteoarthritis and was conducted from May 2024 to November 2025 at the Physical Medicine and Rehabilitation Clinic. Patients who presented to the outpatient clinic with complaints of knee pain and stiffness were examined by the same physiatrist (BCK). Knee osteoarthritis was staged using the Kellgren-Lawrence grading system^16^. Accordingly, patients with knee pain lasting longer than 3 months, a Kellgren and Lawrence score ≥2 on knee radiograph staging, and pain scores between 4 and 8 on the Visual Analog Scale (VAS) were included in the study. Exclusion criteria for the study were: a history of orthopedic surgery of the lower extremity; cognitive dysfunction (Mini-Mental Test score <23); receipt of any treatment for knee osteoarthritis within the last 6 months; an ongoing exercise program; and a history of ligament and ligament injury around the knee.

### Randomization and blinding

Patients were randomized into two groups: a virtual reality-based exercise group (VRG) and a control group, computer-generated random numbers (Excel^©^ 2019), with 1:1 allocation. The randomization procedures were performed by the same physiotherapist (IC). The doctor who conducted the evaluations (SSE) was unaware of the groups. The patients were aware of the study groups.

### Outcome measures

Measurements were taken by the same doctor, who was unaware of group allocations, before treatment and at week 3 (post-treatment). Age, gender, and body mass index were recorded. The VAS and the Western Ontario and McMaster Universities Osteoarthritis Index (WOMAC) were evaluated^17^. A higher WOMAC score is associated with worse functional status. Postural Stability–Dynamic Overall Stability Index (PS-DOSI), Functional Reach Composite Score (FR-CS), Clinical Test of Sensory Interaction on Balance–Composite Score (CTSIB-CS) were recorded using the Biodex Balance System (BBS; Biodex Medical Systems, Inc, Shirley, NY)^18^. Patients removed their shoes before stepping onto the platform for measurements, which were recorded under the supervision of an evaluator. The Short Form-36 was used to assess quality of life^19^.

### Interventions

Patients in the control group received only a conventional rehabilitation program. Patients in the VRG received virtual reality (VR)-based exercise in addition to the conventional rehabilitation program.

#### Conventional rehabilitation program

A traditional rehabilitation program (Transcutaneous Electrical Nerve Stimulation [TENS] Intellect^®^ Advanced [Chattanooga, Mouguerre, France], therapeutic ultrasound, and hot compresses) was applied over a period of 3 weeks, totaling 15 sessions. The TENS was applied to the knee joint with two electrodes via a conventional method. Therapeutic ultrasound was applied for 4 min in continuous mode for deep heating. A hot pack was applied for 20 min as a superficial heating.

#### Virtual reality-based exercise training

The VR-based exercise training was performed following conventional rehabilitation. The same physiotherapist (IC) conducted the VR-based exercise sessions in a dedicated room at the hospital. Each session lasted 30 min. The VR-based exercise training consisted of a total of 15 sessions, held on weekdays over a period of 3 weeks. The exercises selected were Ankle Side Kick, Pumps with Leg Raised, Knee Bends, and Front Knee Exercises. All exercises were performed using the “Microsoft Kinect for Azure” VR software.

### Sample size

The sample size required for the t-test comparing the two groups was calculated using G*Power 3.1.9.7 based on the primary hypothesis concerning differences in balance abilities between the control group and the VRG following the intervention. The sample size was calculated as at least 34 individuals for the two groups, with 95% power and 5% margin of error, and an effect size of d=1.31^20^. Considering the possibility that 50% of participants might drop out during follow-up, 40 participants were initially enrolled.

### Statistical analysis

Statistical analyses were performed using IBM Statistical Package for the Social Science Statistics for Windows, Version 27.0 (IBM Corp., Armonk, NY, USA). Categorical variables were expressed as percentages (%), and continuous variables as mean (standard deviation [SD]). Normality of the data distribution was assessed using visual methods (histograms and probability plots) and analytical tests (Shapiro-Wilk and Kolmogorov-Smirnov). Demographic characteristics of the groups (age, weight, height, and body mass index [BMI]) and continuous variables such as symptom duration and number of comorbidities were compared using the independent-samples t-test or the Mann-Whitney U test depending on their distribution. Categorical variables (gender) were compared using the Chisquare test. For within-group comparisons (before and after intervention), a paired-samples t-test was applied to variables that were normal distributed, and the Wilcoxon signed-rank test was applied to variables that were not normally distributed. A two-way mixed-design repeated-measures analysis of variance (ANOVA) was used to evaluate changes over time (time effect) and differences between groups (group×time interaction). Effect sizes were reported using partial eta squared (η^2^), with η^2^=0.10, 0.25, and 0.40 interpreted as small, medium, and large effect sizes, respectively^21^. The statistical significance level was considered to be p<0.05.

### Ethics approval and consent to participate

The authors obtained permission from the clinical research ethics committee of their affiliated university before commencing the study (2023-19/140). Subsequently, the study was registered at ClinicalTrials.gov before patient recruitment began (NCT06272825). The study was conducted in accordance with the Declaration of Helsinki (1964); informed consent was obtained from the participants.

## RESULTS

The demographic and baseline clinical characteristics of the participants are presented in Table 1. Pre- and post-intervention clinical outcomes of the VRG and control groups are presented in Table 2. Statistically significant improvements were observed in both groups for VAS, WOMAC, the Short Form-36 Physical Functioning (SF-36 PF), PS-DOSI, FR-CS, and CTSIB-CS scores in the post-intervention period (p<0.001). According to the results of the two-way mixed-design repeated-measures ANOVA, the time effect was significant for all parameters (p<0.001). When group-time interaction was examined, significant interactions were observed between the groups in VAS, WOMAC, and SF-36 PF scores (p=0.007, p=0.003, and p<0.001, respectively). The VRG demonstrated significantly greater reductions in pain levels and improvements in disease-specific functional status and overall physical functioning than the control group. Effect sizes were moderate (η^2^=0.117–0.225), with the SF-36 PF subscale showing a higher effect size than those of other parameters.

**Table 1:** Basic characteristics of groups.

	Virtual reality group (n=30)	Conventional group (n=30)	p-value
Gender (male:female)	12:18	11:19	>0.05^c^
Age (years)	67.03 (5.51)	65.04 (5.41)	>0.05^b^
Weight (kg)	76.75 (13.99)	76.12 (11.30)	>0.05^a^
Height (cm)	162.61 (9.02)	162.03 (10.04)	>0.05^a^
BMI (kg/m2)	29.11 (5.13)	29.19 (4.76)	>0.05^a^
Symptom duration (months)	19.93 (15.94)	20.17 (14.97)	>0.05^b^
Number of comorbid diseases	1.73 (1.08)	1.63 (1.03)	>0.05^b^

Notes: ^a^Independent samples t-test;

^b^Mann-Whitney U test;

^c^Chi-square test. BMI: body mass index; kg: kilogram; cm: centimeter; m: meter. Data are presented as mean (standard deviation).

**Table 2:** Clinical outcomes in virtual reality and conventional groups.

Parameters	Virtual reality group (n=30)			Conventional group (n=30)			Time	Group×time	
	Pre-intervention	Post-intervention	p^a/b^	Pre-intervention	Post-intervention	p^a/b^	p^c^	F/p^c^	η^2^
VAS	6.86 (1.04)	3.06 (1.05)	<0.001^a^	7.00 (1.08)	3.90 (1.12)	<0.001^a^	p<0.001	7.681/0.007	0.117
WOMAC	38.53 (9.43)	24.21 (7.73)	<0.001^b^	38.13 (8.18)	26.00 (7.91)	<0.001^b^	p<0.001	9.699/0.003	0.143
SF-36 PF	32.66 (12.69)	47.76 (12.31)	<0.001^b^	31.53 (12.60)	44.06 (12.09)	<0.001^a^	p<0.001	16.849/<0.001	0.225
PS-DOSI	2.46 (0.81)	1.59 (0.78)	<0.001^a^	2.41 (0.76)	1.71 (0.73)	<0.001^a^	p<0.001	1.951/0.168	0.033
FR-CS	18.27 (3.69)	13.48 (2.93)	<0.001^b^	18.86 (3.66)	14.75 (3.30)	<0.001^b^	p<0.001	1.702/0.197	0.029
CTSIB-CS	4.17 (1.85)	2.37 (1.14)	<0.001^a^	4.39 (1.97)	3.14 (1.49)	<0.001^a^	p<0.001	2.662/0.108	0.044

Notes: p^a^: Wilcoxon signed-rank test;

p^b^: Paired-samples t-test;

p^c^: Two-way mixed-design repeated-measures analysis of variance; η^2^=Partial eta squared; Values are expressed as mean (SD); p<0.05. VAS: visual analog scale; WOMAC: Western Ontario and McMaster Universities Osteoarthritis Index; SF-36 PF: Short Form-36 Physical Functioning; PS-DOSI: Postural Stability–Dynamic Overall Stability Index; FR-CS: Functional Reach Composite Score; CTSIB-CS: Clinical Test of Sensory Interaction on Balance–Composite Score.

Pre- and post-intervention results for the SF-36 sub-dimensions of the VRG and control groups are presented in Table 3. In both groups, statistically significant improvements were observed in all sub-dimensions of the SF-36 (Physical Functioning, Role Physical, Bodily Pain, General Health, Vitality, Social Functioning, Role Emotional, and Mental Health) in the post-intervention period (p<0.001). According to two-way mixed-methods repeated-measures ANOVA analysis, the time effect was significant in all SF-36 subdimensions (p<0.001). When the group-by-time interaction was examined, statistically significant interactions were observed for the subdimensions Physical Functioning, Bodily Pain, General Health, Vitality, Social Functioning, and Role-Emotional (p<0.05). These findings indicate that the improvement observed in VRG for these sub-dimensions was statistically significantly greater than in the control group. In the Role Physical and Mental Health sub-dimensions, no significant group-by-time interaction was found (p=0.151 and p=0.604, respectively). Improvements in these sub-dimensions were found to be at a similar level in both groups. Effect sizes range from small to medium to moderate (η^2^=0.005–0.293). The subdimensions of Bodily Pain, Vitality, and Physical Functioning exhibited larger effect sizes than other parameters.

**Table 3:** SF-36 outcomes in the virtual reality and conventional groups.

SF-36 Parameters	Virtual reality group (n=30)			Conventional group (n=30)			Time	Group×time	
	Pre-intervention	Post-intervention	p^a/b^	Pre-intervention	Post-intervention	p^a/b^	p^c^	F/p^c^	η^2^
Physical functioning	32.66 (12.69)	47.76 (12.31)	<0.001^b^	31.53 (12.60)	44.06 (12.09)	<0.001^a^	p<0.001	16.849/<0.001	0.225
Role physical	16.20 (10.16)	31.66 (9.88)	<0.001^a^	14.86 (9.97)	28.53 (8.81)	<0.001^a^	p<0.001	2.113/0.151	0.035
Bodily pain	27.66 (8.81)	43.86 (8.93)	<0.001^a^	27.36 (8.19)	39.60 (8.88)	<0.001^a^	p<0.001	24.008/<0.001	0.293
General health	44.06 (14.37)	59.40 (14.32)	<0.001^a^	43.30 (14.18)	55.16 (14.19)	<0.001^a^	p<0.001	13.541/0.001	0.189
Vitality	33.43 (11.49)	50.20 (10.40)	<0.001^b^	32.56 (10.79)	45.56 (10.25)	<0.001^b^	p<0.001	21.528/<0.001	0.271
Social functioning	43.56 (17.51)	60.06 (16.74)	<0.001^a^	42.50 (16.48)	55.43 (16.60)	<0.001^a^	p<0.001	15.780/<0.001	0.214
Role emotional	39.53 (13.42)	54.96 (13.25)	<0.001^a^	37.50 (13.02)	50.33 (13.14)	<0.001^a^	p<0.001	9.976/0.003	0.147
Mental health	51.50 (13.23)	67.96 (13.08)	<0.001^a^	50.50 (12.49)	67.40 (13.31)	<0.001^a^	p<0.001	0.271/0.604	0.005

Notes: p^a^: Wilcoxon signed-rank test;

p^b^: Paired-samples t-test;

p^c^: Two-way mixed-design repeated-measures analysis of variance; η^2^=Partial eta squared; Values are expressed as mean (SD); p<0.05. SF-36: Short Form-36.

## DISCUSSION

According to the results of this study, incorporating virtual reality-based exercise therapy into the rehabilitation of patients with symptomatic knee osteoarthritis leads to greater reductions in pain levels and improvements in disease-specific functional status, overall physical function, postural stability, dynamic balance, and sensory integration. In addition, virtual reality-based exercise therapy has resulted in improvements in quality-of-life parameters such as physical functioning, bodily pain, general health, social functioning, vitality, and role-emotional. This improvement may be related to the fact that virtual reality exercises and games allowing individuals to detach from the hospital environment while exercising, forgetting their role as a patient, and overcoming kinesiophobia by immersing themselves in the games. Virtual reality exercises did not provide any additional benefit to the physical role limitations and mental health aspects of quality of life. To the authors’ knowledge, this study is the first in the literature to evaluate the effectiveness of virtual reality-based exercise therapy on quality of life in patients with knee osteoarthritis.

It has been reported that adding a total of 30 sessions of non-immersive virtual reality-based exercise therapy to conventional rehabilitation resulted in an improvement in pain among patients with knee and hip osteoarthritis^22^. Similarly, this study also found that virtual reality-based exercise therapy resulted in improvements in both pain intensity and the bodily pain subscale of quality of life. Researchers have reported that immersive virtual reality exercises are effective in reducing pain and improving function in patients with knee osteoarthritis. Literature reports indicate that treatment adherence is initially high, but adherence to virtual reality-based exercise therapy decreases over time^23^. Although this study employed non-immersive virtual reality exercises, it found that incorporating these exercises into traditional treatment resulted in greater improvement in pain and disease-specific functional limitations, and the physical-function domain of quality of life.

One study reported that non-immersive virtual reality-based exercise therapy was effective in improving static and dynamic balance and proprioception, as assessed by the Timed Up and Go test, and in improving independence in activities of daily living and in reducing pain in patients with knee osteoarthritis^24^. Another recent study reported that immersive virtual reality-based exercises, when added to conventional treatment, had a positive effect on pain, function, and balance in patients with knee osteoarthritis. In the aforementioned study, balance was assessed using the Berg Balance Scale^15^. To the authors’ knowledge, this study is the first in the literature to evaluate the effectiveness of virtual reality exercises on balance in patients with knee osteoarthritis using a device. According to the results of this study, the addition of virtual reality exercises resulted in improvements in postural stability, dynamic balance, and functional stability, and furthermore, sensory integration, which assesses the somatosensory and vestibular systems. These findings are consistent with the literature and demonstrate that virtual reality-based exercise therapy may effectively achieve holistic improvement in balance among patients with knee osteoarthritis. However, we believe that further studies are needed to determine efficacy with respect to balance and proprioception in this patient population.

In a recent study, virtual-reality headsets were used for immersive therapy, and patients reported temporary side effects such as mild nausea and transient headaches^15^. In this study, virtual reality exercises were performed non-immersively, and no adverse effects were reported by the participants.

### Limitations

That this study reports short-term efficacy can be considered a limitation. Although 1-month follow-up examinations were planned, we were unable to reach a sufficient number of patients. Studies evaluating long-term effectiveness will contribute to the literature. However, the strength of this study lies in its single-blind design, its relatively quantitative and comprehensive evaluation parameters, and its supervision of the virtual reality exercises by a physiotherapist.

## CONCLUSION

Adding virtual reality-based exercises to the rehabilitation in patients with symptomatic knee osteoarthritis leads to greater reductions in pain levels and improvements in disease-specific functional status, overall physical function, postural stability, dynamic balance, and sensory integration. Furthermore, the addition of virtual reality-based exercises further improved quality of life across all subdomains except for physical role limitations and mental health.

## Data Availability

The datasets generated and/or analyzed during the current study are available from the corresponding author upon reasonable request.
